# A decade of change: performance trajectories in Paralympic discus throw across classes and genders (2012–2022)

**DOI:** 10.3389/fspor.2026.1821664

**Published:** 2026-07-02

**Authors:** Luiz Gustavo T. F. Santos, Alex José Sabino, João Paulo Casteleti de Souza, João Paulo Cunha, Ciro Winckler

**Affiliations:** 1 Sports Development Department, Brazilian Paralympic Committee, São Paulo, Brazil; 2 High-Performance Department, Brazilian Paralympic Committee, São Paulo, Brazil; 3 Instituto Elisangela Maria Adriano - IEMA, São Caetano, São Paulo, Brazil; 4 Parasport Study Center, São Paulo Federal University, Santos, Brazil

**Keywords:** classification system, longitudinal analysis, Para athletics, Paralympic, performance trends

## Abstract

**Introduction:**

A longitudinal analysis of performance trajectories in Paralympic discus throw events from 2012 to 2022, focusing on gender and classification-specific trends.

**Methods:**

Using annual rankings from the International Paralympic Committee, we examined the top 1–3 and top 4–8 athletes in each class to assess technical evolution, participation dynamics, and the impact of external disruptions such as the COVID-19 pandemic.

**Results:**

Heterogeneous performance patterns were revealed, with some classes showing sustained improvement while others experienced regression. Notably, the F11 class has shown consistent growth in performance results, suggesting strategic potential for national investment for countries seeking to invest in World Championship events.

**Discussion:**

The findings underscore the importance of classification-based monitoring and evidence-informed planning for athlete development, talent identification, and competition optimization. These insights contribute to the refinement of training strategies and support decision-making by coaches and Paralympic committees.

## 1 Introduction

Para athletics has been part of the Paralympic Games since its 1st edition in Rome in 1960, and it was also present in the International Stoke Mandeville Games in 1953. At the Paralympic Games of Tokyo 2020, Paris 2024, and Los Angeles 2028, Para athletics maintained its significance, with over 160 gold medals contested in each edition. Specifically in the discus throw, 12 gold medals were awarded to athletes with physical and visual impairments, highlighting the consistency of the event across Paralympic cycles (1–4). The eligible classes, such as F37, F38, F41, F51, F52, F53, F55, F57, F64, and F11^1^, reflect the functional diversity of the participants. In 2028, the inclusion of class F51 indicates ongoing adjustments in the functional classification system over the years. The number of events is determined by the Paralympic Classification System, which ensures fair competition by grouping athletes based on their impairment and sports functionality (3, 4).

**Figure 1:**
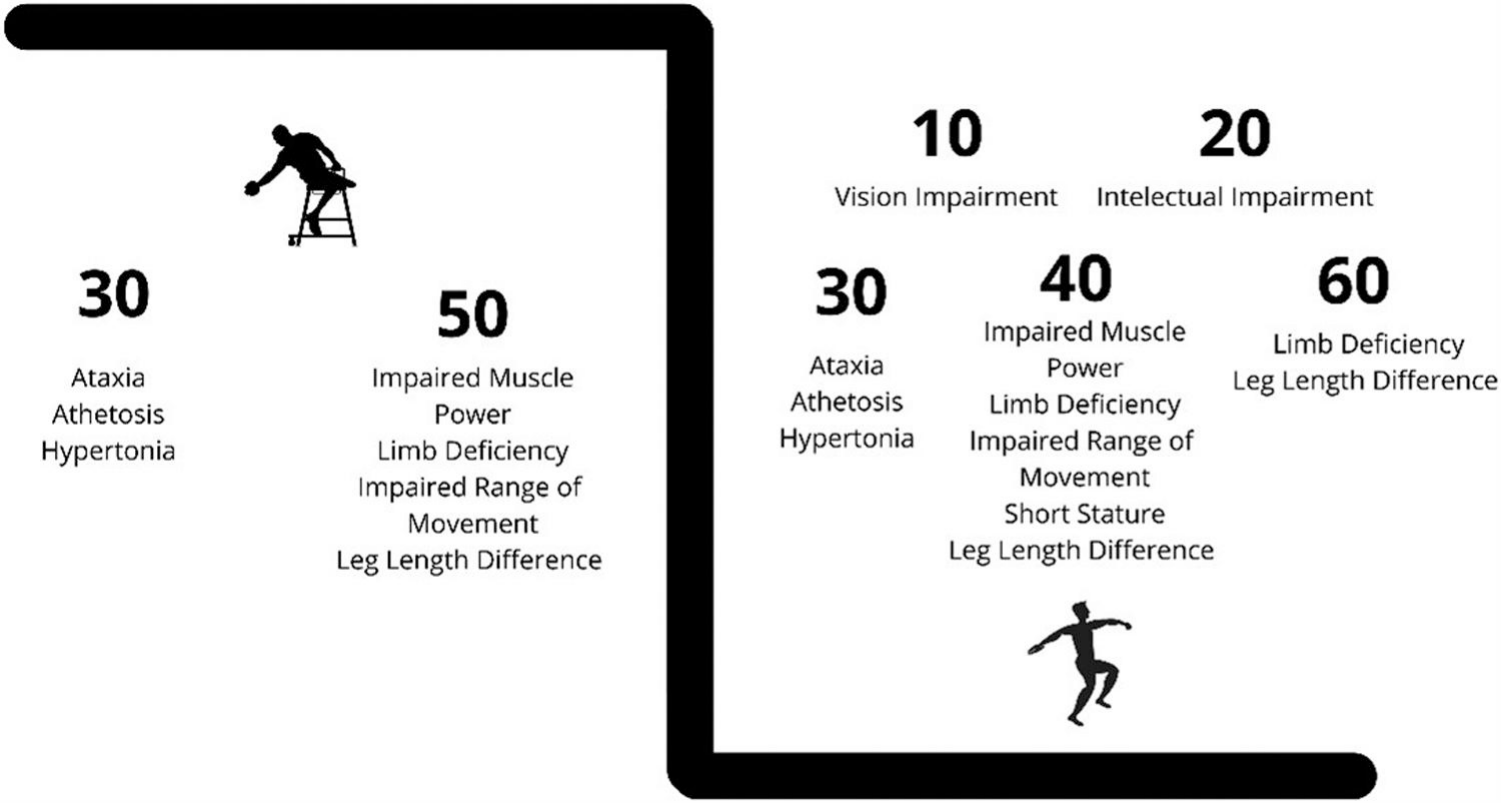
Profile of the classes in Discus Throw Events.

In standing classes (F11, F37, F38, F41, and F64), athletes compete based on the weight of the implement used, which varies across male classes by function and sex (5, 6). In seated classes (F52, F53, F55, F56, and F57), all athletes compete sitting on a bench with a minimum height and width specified by the rules, and the implement used in these classes is the same for both sexes (Figure 1) (6). In the standing classes, where athletes compete on their feet, using speed as a physical and technical factor is fundamental for high-performance competition (7–10). Due to this characteristic, the spinning technique is observed in some classes as a resource to optimize performance, which is not observed in the seated classes where athletes compete using the bench (6).

In Paralympic sports, this theme needs to be explored due to the type and cause of impairment. Dehghansai et al. (11) stand out as variables that can facilitate or hinder Paralympic athletes’ access to the elite performance level. Among them is the Paralympic Classification System. In Para athletics, as well as other Paralympic sports, the impact of changes in rules and adaptations used by competitors is perceived. To get the best results, coaches and athletes could develop cycle periodization based on the International World Ranking, World Championships, or Paralympic Cycle marks. Current studies aim to longitudinally observe the evolution of certain Paralympic Field events in athletics, providing reference values for coaches and sports managers to inform decision-making related to changes in training and economic planning, respectively (5, 12). Monitoring the evolution of Paralympic events can be one of the strategies used by National Paralympic Committees to select athletes with potential for international medals and thus improve their position in the Paralympic Games medal table.

Planning an athlete’s career to ensure the evolution of sports brands is the responsibility of the coach and their multidisciplinary team. In some Olympic sports, the age at which an athlete can achieve their best marks is already known. In athletics, the throwing events show the most significant changes when analyzing yearly performance in relation to other events. Furthermore, women’s expressive development compared to men in these events (13) is notable. Tracking the sporting results of a given event is a fundamental tool for defining the strategies and goals of the coach and their athlete, not only to establish performance targets but also to organize training and competitions (14). Studies are characterized as cross-sectional or longitudinal in the analysis of results. Measures can focus on the peak performance age relationship (15–17), sex (18), equipment influence (19), Paralympic functional classification (19) and performance evolution through the years (9). Athletes with acquired disabilities classified to compete in the lower classes have been more frequently seen among the top three highlights because their structure, even after the disability, provides them with certain advantages. These advantages include a muscular structure and access to specialized training, even under adverse conditions, allowing them to achieve high performance at an advanced age. This development process would not be possible in some Olympic sports (20).

This study aimed to identify the evolution in discus throw events in the International Paralympic Committee Annual Ranking-IPC (2012–2022) to descriptive differences and athletic performance by the level of men and women top ranking athletes (N^o^. 1 to N^o^. 8) who participated in discus throw events by class and provide the relevant information to contribute to the improvement of field Para athletics’ performance. From the structuring of the results information during the selected period, three questions guide the development of this research: i)Has there been an increase in the number of National Paralympic Committees and athletes, according to the gender, competed in Discus Throw events according to the respective sport class? ii) How did the results for each class behave in the last decade for the Discus Throw event for TOP_1−3_ and TOP_4−8_? iii) Which events have evolved in the last decade, and what is the evolution (%) accumulated for TOP_1−3_?.

## 2 Methods

### 2.1 Data sources

This study used a retrospective cohort design, analyzing officially ratified discus throw performance data from World Para Athletics–sanctioned competitions. A total of 335 athletes, unique para-athletes using their athlete identification number (SDMS) were analyzed (137 in the men’s classes and 198 in the women’s classes) annual rankings were collected: 2012 (92 pieces of data, 40 in the men’s classes and 52 in the women’s classes); 2013 (94 pieces of data, 40 in the men’s classes and 54 in the women’s classes); 2014 to 2019, 2021 to 2022 (96 pieces of data, 40 in the men’s classes and 56 in the women’s classes each per year), and 2020 (91 pieces of data, 40 in the men’s classes and 51 in the women’s classes).

For the years 2012 and 2013, the following events were excluded from the database: Men’s F52, Men’s F56, Women’s F41, Women’s F52, Women’s F55, and Women’s F57. These exclusions were necessary due to substantial modifications in competition rules during this period, particularly concerning the dimensions, positioning, and configuration of the throwing benches (F52–F57), as well as adjustments in the functional classification criteria (F41).These rules were primarily implemented to standardize the equipment used in competition and ensure equitable conditions for athletes. However, they also resulted in meaningful alterations in the technical execution patterns of the throwing movements. Consequently, performance outcomes from these years are not directly comparable with subsequent periods, thereby justifying their exclusion from the longitudinal analysis.

To achieve the purpose of this study, it identified changes in the TOP_1−3_ and TOP_4−8 events_, consecutively carried out by class, in the Discus Throw (Meńs classes=5 and Womeńs classes=7) events during 2012-2022, according to the Paris 2024 program. For annual ranking records, results provided by the IPC (http://www.paralympic.org) were collected (21); namely, the data were collected based on the athletes’ records of best performance by year. As shown in Table 2, the *p*-value difference between the TOP 3 and TOP 8.

### 2.2 Data analysis process

The research variable was divided into men’s and women’s classes in the Top 3 (*n* = 3 athletes) and Top 8 (*n* = 5 athletes) on discus throw by class each year. Changes in performance were analyzed based on the TOP_1−3_ and TOP_4−8 events_ rankings for each class. TOP_1−3_ and TOP_4−8 events_ represent the three best performances of the year, while the TOP_1−3_ and TOP_4−8 events_ include the fourth to eighth best performances of the year. In cases where the same athlete appeared more than once within the rankings, only the best performance was considered. This approach ensured that only three distinct athletes were included in the TOP_1−3_ events and five distinct athletes in the TOP_4−8 events._ The best annual mark of each athlete in their respective class was used to determine the mean value and standard deviation per class. This approach ensured that only three distinct athletes were included in the TOP_1−3_ events and five distinct athletes in the TOP_4−8 events._The % accumulated (%Ac) was calculated from the sum of the annual evolution of each (%AE). All data were analyzed by Microsoft® Excel (22, 23).

Variables Definitions:

%AE=(100*MeanValue2013)/(MeanValue2012)−100

%Ac=%AE2013/2012+%AE2014/2013+……%AE2022/2021

### 2.3 Statistical analysis

An inferential comparative statistical analysis was conducted on the results, (Avg ± sd) across the analyzed groups. The factors considered included performance categories (Top), sport classes, and years of observation. Comparisons were based on estimates of differences between Avg, accompanied by their respective 95% confidence intervals (95% CIs). To identify statistically significant differences among groups, ANOVA-Test was applied. Significant effects were detected, and *post hoc* analyses using Tukey’s test. The significance level was *α* = 0.05, and all statistical analyses were conducted using GraphPad Prism.

## 3 Results

**Figure 2:**
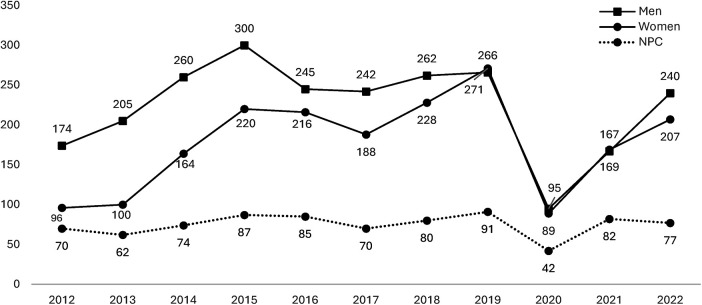
Quantification of the number of men, women, and National Paralympic Committees.

Figure 2 shows the number of male and female athletes and National Paralympic Committees (NPĆs) participating in the Discus throw events from 2012 to 2022. The highest number of participants was identified in 2019 (men 266 and women 271) distributed at 91 NPĆs with the highest number of women participating in the implement and the second highest number of men.

In Table 1, the values of results (Avg ± Sd) and %Ac for the male events were presented. It was observed that among the Top 3 performances, the F37 class was the only event that showed a regression in technical level (%Ac = −1.05). The F64 class had the lowest improvement (%Ac = 0.73), followed by the F56 class (%Ac = 1.77). Examining the TOP 8 performances, the F56 class (%Ac = −0.93) showed a technical regression. The F11 class (%Ac = 7.46) exhibited the least improvement, followed by the F37 class (%Ac = 7.67). Comparing 2012 to 2013, 2013 to 2014, and so on, the following significant differences were found: In **F37-M** (Top 3: 2020 vs. 2021: *p* = 0,0288; Top8: 2014 vs. 2015:*p* = 0,0232; 2016 vs. 2017:*p* = 0,0126; 2018 vs. 2019:*p* = 0,0444; 2019 vs. 2020:*p* = 0,0006; 2020 vs. 2021:*p* = <0,0001). In **F52-M** (Top8: 2012 vs. 2013:*p* = 0,0303; 2015 vs. 2016:*p* = 0,0472; 2016 vs. 2017:*p* = 0,0084; 2017 vs. 2018:*p* = 0,004; 2019 vs. 2020:*p* = 0,0132; 2020 vs. 2021:*p* = 0,0136). In **F56-M** (Top3: 2019 vs. 2020:*p* = 0,0457; 2020 vs. 2021:*p* = 0,0354; Top8: 2015 vs. 2016:*p* = 0,0155; 2017 vs. 2018:*p* = 0,0116; 2018 vs. 2019:*p* = 0,0336; 2019 vs. 2020:*p* = 0,0002; 2020 vs. 2021:*p* = 0,0005. In **F64-M** (Top3: 2020 vs. 2021:*p* = 0,0294; 2021 vs. 2022:*p* = 0,0282; Top8: 2015 vs. 2016:*p* = 0,0274; 2019 vs. 2020:*p* = 0,0237; 2020 vs. 2021:*p* = 0,0255).

**Table 1:** Men’s events differences betweenTOP3 and TOP8 by class in the discus throw during 2012 to 2022.

Class	TOP	%Ac	2012	2013	2014	2015	2016	2017	2018	2019	2020	2021	2022	*p*-Value
F11	TOP3	15,52	37,69 (±0,63)	37,39 (±1,70)	38,04 (±1,59)	39,09 (±0,94)	41,43 (±2,49)	41,63 (±3,99)	44,65 (±1,99)	43,95 (±1,97)	40,62 (±3,61)	42,85 (±2,71)	43,60 (±2,70)	0,0031
	TOP8	7,46	35,44 (±0,77)	34,3 (±1,10)	35,65 (±0,24)	35,94 (±0,74)	36,59 (±0,81)	35,00 (±1,86)	36,62 (±0,54)	37,53 (±0,63)	31,43 (±3,96)	36,42 (±0,73)	37,05 (±1,74)	
F37	TOP3	−1,05	53,96 (±1,88)	53,39 (±2,02)	53,65 (±1,35)	53,32 (±1,00)	55,59 (±3,60)	54,43 (±0,73)	50,05 (±1,78)	52,27 (±0,73)	46,37 (±1,64)	54,52* (±0,64)	51,95 (±2,03)	0,0112
	TOP8	7,67	47,06 (±2,85)	48,02 (±1,65)	48,28 (±1,97)	50,05* (±1,60)	50,02 (±2,51)	47,51* (±2,97)	47,36 (±0,82)	49,55* (±1,65)	40,77*** (±2,42)	48,77*** (±2,70)	48,91 (±1,86)	
F52	TOP3	20,46	-	-	19,98 (±2,03)	18,89 (±2,21)	20,00 (±1,28)	21,68 (±2,27)	20,16 (±0,43)	20,85 (±0,82)	20,69 (±0,47)	20,35 (±0,45)	20,13 (±0,16)	0,0052
	TOP8	41,91	-	-	13,53 (±1,08)	14,81 (±0,86)	15,94* (±0,79)	17,21** (±0,70)	18,50** (±0,91)	18,53 (±0,90)	15,62* (±1,62)	18,51* (±1,29)	18,53 (±1,25)	
F56	TOP3	1,77	-	-	42,61 (±2,54)	43,00 (±3,52)	44,76 (±0,66)	45,17 (±0,43)	45,69 (±0,97)	44,63 (±1,21)	36,94* (±2,45)	44,60* (±1,37)	45,39 (±3,14)	0,0028
	TOP8	−0,93	-	-	36,43 (±0,28)	37,60 (±1,18)	39,54* (±0,88)	40,19 (±1,73)	39,23* (±1,65)	40,55* (±1,41)	28,47*** (±1,45)	39,62*** (±2,47)	39,25 (±1,28)	
F64	TOP3	0,73	60,29 (±2,77)	58,81 (±3,75)	61,35 (±0,87)	61,57 (±1,32)	63,07 (±1,23)	61,42 (±2,92)	63,81 (±1,43)	62,92 (±0,79)	54,53 (±3,76)	63,59* (±2,66)	59,08* (±2,25)	0,0044
	TOP8	13,18	48,15 (±3,51)	45,56 (±2,37)	50,38 (±4,92)	50,51 (±4,49)	54,31* (±4,66)	54,53 (±2,26)	54,19 (±3,43)	54,61 (±3,17)	45,34* (±4,41)	54,17* (±2,72)	52,62 (±2,57)	

* *p* < 0.05, ** *p* < 0.01, *** *p* < 0.001.

In Table 2, the values of results (Avg ± sd) and %Ac for the female events are presented. It was observed that among the TOP 3 performances, the F11 class had the lowest improvement (%Ac = 2.07), followed by the F38 (%Ac = 5.56) and F57 (%Ac = 21.47). Examining the TOP 8 performances, the F38 class (%Ac = 1.81) exhibited the least improvement, followed by the F57 (%Ac = 28.21) and F55 (%Ac = 59.51). Comparing 2012 between 2013, 2013 between 2014, and so on, the following significant differences were found: In F11-W (Top3: 2019 vs. 2020: *p* = 0,0359; 2020 vs. 2021:*p* = 0,0359; Top8: 2012 vs. 2013:*p* = 0,0053; 2013 vs. 2014:*p* = 0,0271; 2015 vs. 2016:*p* = 0,0459; 2018 vs. 2019:*p* = 0,0222). In F38-W (Top3: 2020 vs. 2021:*p* = 0,0361; 2021 vs. 2022:*p* = 0,0156; Top8: 2014 vs. 2015:*p* = 0,0187; 2019 vs. 2020:*p* = 0,0037; 2020 vs. 2021:*p* = 0,0089; 2021 vs. 2022:*p* = 0,011). In F41-W (Top3: 2021 vs. 2022:*p* = 0,027; Top 8: 2015 vs. 2016:*p* = 0,0002; 2018 vs. 2019:*p* = 0,0224; 2019 vs. 2020:*p* = 0,0254; 2020 vs. 2021:*p* = 0,0003). In F53-W (Top3: 2020 vs. 2021:*p* = 0,042; Top8: 2012 vs. 2013:*p* = 0,0219; 2014 vs. 2015:*p* = 0,0137; 2015 vs. 2016:*p* = 0,0009). In F55-W (Top3: 2014 vs. 2015:*p* = 0,0234; 2020 vs. 2021:*p* = 0,0082; Top8: 2013 vs. 2014:*p* = 0,0006; 2014 vs. 2015:*p* = 0,002; 2015 vs. 2016:*p* = 0,0209; 2019 vs. 2020:*p* = 0,0013; 2020 vs. 2021:*p* = 0,0002). In F64-W (Top8: 2013 vs. 2014:*p* = 0,0016; 2014 vs. 2015:*p* = 0,0015; 2020 vs. 2021:*p* = 0,0031).

**Table 2:** Women’s events differences between TOP3 and TOP8 by class in the discus throw during 2012 to 2022.

Class	TOP	%Ac	2012	2013	2014	2015	2016	2017	2018	2019	2020	2021	2022	*p*-Value
F11	TOP3	2,07	37,59 (±4,22)	32,15 (±5,20)	33,27 (±4,98)	34,15 (±2,85)	35,30 (±2,53)	34,28 (±0,76)	34,48 (±1,85)	36,66 (±1,29)	31,53 (±0,70)	36,11 (±0,50)	37,01 (±1,70)	0,0022
	TOP8	58,78	20,82 (±1,22)	21,68** (±1,38)	25,5* (±1,77)	26,54* (±2,16)	29,41 (±2,29)	29,10 (±2,67)	29,56 (±2,59)	32,38* (±2,63)	18,87 (±2,69)	32,42 (±1,30)	26,58* (±4,27)	
F38	TOP3	5,56	32,81 (±2,27)	31,02 (±1,82)	30,41 (±1,02)	31,99 (±1,45)	34,55 (±2,79)	32,79 (±2,04)	33,85 (±2,80)	36,57 (±2,33)	32,35 (±2,04)	37,32* (±1,51)	33,51* (±1,3)	0,0108
	TOP8	1,81	28,37 (±0,97)	27,90 (±1,47)	28,66 (±0,73)	29,80* (±0,44)	30,48 (±1,01)	29,41 (±0,94)	28,48 (±1,78)	30,81 (±2,59)	27,51** (±2,53)	33,14** (±1,79)	27,58* (±1,37)	
F41	TOP3	34,55	-	-	25,64 (±2,76)	26,28 (±3,01)	29,23 (±3,59)	29,08 (±2,94)	31,49 (±3,91)	33,55 (±1,15)	28,88 (±2,87)	35,48 (±3,73)	34,62* (±3,64)	0,0050
	TOP8	61,56	-	-	16,36 (±4,53)	21,46 (±1,24)	24,64*** (±1,11)	24,60 (±1,62)	25,01 (±1,47)	27,56* (±1,15)	23,10* (±2,25)	28,11*** (±1,81)	27,65 (±1,09)	
F53	TOP3	47,48	-	-	10,86 (±1,76)	11,89 (±0,97)	12,87 (±0,70)	12,52 (±0,65)	14,93 (±1,89)	15,56 (±1,79)	9,84 (±0,49)	15,99* (±1,44)	15,71 (±2,25)	0,0046
	TOP8	72,39	-	-	8,03 (±0,80)	9,90 (±0,56)	10,29 (±0,58)	10,48 (±0,82)	11,13 (±1,01)	12,30 (±0,78)	5,62 (±3,42)	12,53 (±1,07)	10,88 (±0,82)	
F55	TOP3	25,62	-	-	22,24 (±2,27)	22,95* (±2,35)	24,13 (±1,28)	22,56 (±0,65)	23,69 (±0,52)	23,75 (±0,69)	21,54 (±1,11)	25,96 (±0,84)	24,96 (±2,00)	0,0052
	TOP8	59,51	-	-	17,21 (±0,59)	19,03** (±0,38)	19,97* (±0,56)	19,58 (±1,04)	20,57 (±1,35)	21,88 (±0,81)	16,44** (±1,46)	22,54*** (±1,39)	21,03 (±1,19)	
F64	TOP3	52,48	24,78 (±0,65)	26,33 (±2,13)	35,78 (±6,06)	38,59 (±6,84)	40,16 (±6,68)	38,55 (±1,06)	37,43 (±1,40)	37,84 (±2,30)	35,61 (±1,80)	41,02 (±3,47)	38,94 (±0,44)	0,0042
	TOP8	62,68	18,56 (±2,26)	21,69 (±1,69)	26,43** (±1,39)	29,00** (±1,38)	29,59 (±1,69)	30,45 (±1,58)	31,18 (±2,22)	32,88 (±1,87)	29,72 (±2,11)	35,44** (±1,69)	32,54 (±3,09)	

* *p* < 0.05, ** *p* < 0.01, *** *p* < 0.001.

## 4 Discussion

**Figure 3:**
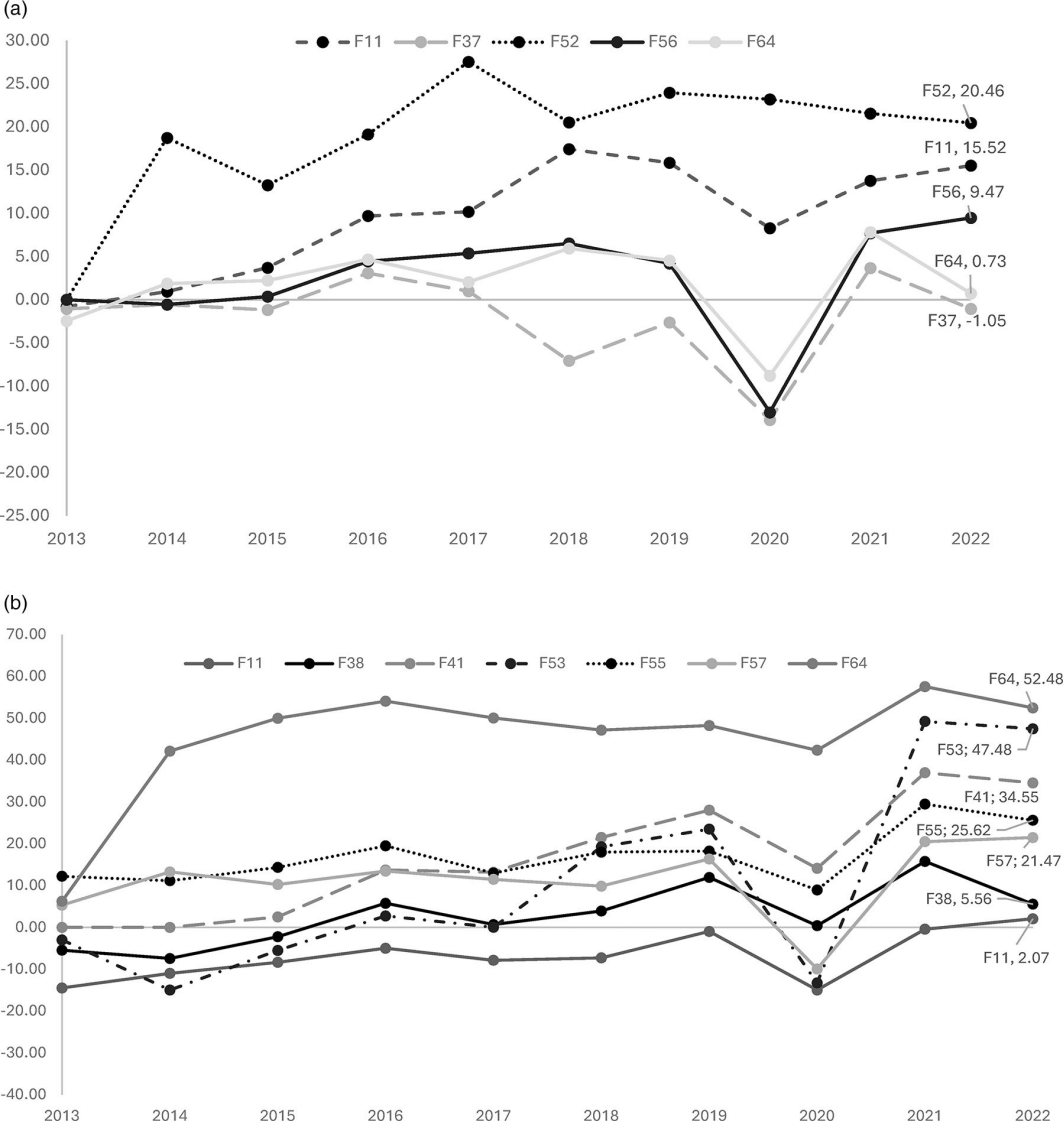
Men's paralympic events TOP3 % of performance evolution change by Class (**a**) and Women's paralympic events TOP3 % of performance evolution change by Class (**b**).

This study was conducted to provide information contributing to the improvement of Para athletics’ performance by identifying changes in the World Ranking classes for Discus Throw events in the IPC (2012–2022) and assessing athletic performance among the top-ranked male and female athletes (No. 1 to No. 8). Currently, in the scientific literature (24–26), understanding the pathway of athlete retention in Paralympic sport until retirement and the main variables influencing this process can optimize the actions taken by Paralympic committees. Sports stakeholders must use technical information to support administrative decisions that affect the careers of athletes and coaches. In this context, understanding performance trends through structured monitoring of international rankings is essential for refining recruitment strategies, optimizing training loads, and enhancing competition planning (27). Results from the last decade (Figure 3) can be an essential tool for training, attracting new athletes, and determining strategic competitions to maintain the world ranking.

### 4.1 National Paralympic committees and athletes’ participations

The highest number of participants was recorded in 2019 (men=266 and women=271), distributed across 91 NPCs. This represented the highest number of women participating in the event and the second-highest number of men. The continental events held in 2019 may have contributed to the high participation rates, particularly for entries to the Tokyo 2020 Paralympic Games. In 2020, the suspension of the Tokyo Games and the World Health Organization’s (2020) announcement of the COVID-19 pandemic led some countries to suspend international events that validate performances for world rankings, thereby affecting the number of registered athletes on the international stage. Despite the resumption of some international events in 2021 and 2022, it has not been possible to date to restore the level of participation by athletes and Paralympic committees observed in 2019. The observed figures have remained like those of 2016–2018.

This reduction in participation reflects a global impact on competitive structures and access to training during the pandemic. According to Marmeleira (28), interruptions to preparation periods and the closure of adapted training facilities particularly affected athletes in technical and field-based events, compromising their readiness for peak performance.

The long-term impact of pandemic interruptions should also be interpreted cautiously, given that historical data on master’s athletes (9) shows consistent improvements in throwing events over decades, even in older age groups. This resilience may inspire similar expectations for post-pandemic recovery among para-athletes. Moreover, performance recovery in Para athletics has previously demonstrated robust patterns in the face of adversity, with identifying sustained improvement trends in sprint events across four Paralympic cycles (27) Therefore, although pandemic effects were significant in 2020, the resumption of competitions and the proximity to the Tokyo Paralympic cycle may partially explain the performance improvements observed in certain classes from 2021 onwards.

### 4.2 Men’s event

The results for the meńs classes showed significant differences for the Top 1–3 and Top 4–8 groups and classes. All classes worsened their international ranking marks when comparing 2020 to 2019, with a significant difference in the TOP1–3 for class F56 (*p* < 0.05) and in the TOP8 for classes F37 (*p* < 0.001), F52 (*p* < 0.05), F56 (*p* < 0.001), and F64 (*p* < 0.05). This decline in performance may hypothetically be associated with the pandemic period and the resulting interruption of international competitions and training schedules, as well as with the official guidelines issued by the World Health Organization during 2020. Marmeleira (28) observed that disruptions related to the COVID-19 pandemic substantially affected athletes’ preparation across Paralympic events, especially in disciplines with greater technical complexity and limited access to training facilities, such as field events. Another potential associated factor is that in 2019, the World Championship and continental qualifying events for the Tokyo 2020 Paralympic Games took place, which increased the mark values.

Between 2021 and 2020, a significant difference and improvement in mark value were observed for the TOP1–3 group in classes F37 (*p* < 0.001) and F56 (*p* < 0.001). Such improvement aligns with longitudinal analyses of throwing events, which demonstrate cyclical performance patterns influenced by competitive calendars and training availability, following a cubic trend over time (29). In line with Frossard (8), these fluctuations also reflect how inter-class performance dispersion varies with differences in equipment adaptation, competition density, and athletes’ functional variability.

It is worth noting that class F37 was the only one among the TOP1–3 that showed a negative accumulated evolution (%Ac = –1.05) for the period 2012–2022, making it a class that should be strategically observed by NPCs for potential gold medal conquest. This trend may reflect structural limitations in talent development and retention, especially in events with greater technical demands. As reported by Piacentini et al. (30), field events, particularly in the throwing disciplines, exhibit higher dropout rates among junior athletes due to early specialization, biomechanical complexity, and limited continuity of support during transition phases.

This variability highlights the importance of evidence-based monitoring of international performance dispersion across classes, as suggested by Frossard (8), particularly to ensure that classification processes support fair competition and performance potential across different functional profiles. Additionally, studies in paralympic sport emphasize that individual biomechanical adjustments may disproportionately influence performance in classes with asymmetrical impairments, further complicating standardized performance trajectories (31–33).

Despite the COVID-19 pandemic, significant fluctuations in international marks were observed during the analyzed period, reinforcing the role of competition level, classification-based variability, and calendar structure in shaping performance trends (8, 9, 29).

### 4.3 Women’s event

The results observed for the women’s classes, similar to those for the men’s, showed differences between the TOP1–3 and TOP4–8 groups and classes. For the period from 2020 to 2019, it was noted that all classes demonstrated a reduction in international ranking marks, although significant differences were evident only for the TOP4–8 group in classes F38 (*p* < 0.01), F41 (*p* < 0.05), and F55 (*p* < 0.01). For the period from 2021 to 2020, all classes exhibited significant improvements in the marks recorded in the international ranking, with the TOP1–3 group showing progress in classes F38 (*p* < 0.05) and F53 (*p* < 0.05), and the TOP4–8 group exhibiting gains in classes F38 (*p* < 0.01), F41 (*p* < 0.001), F55 (*p* < 0.001), and F64 (*p* < 0.01). This evolution suggests a rebound effect following the competitive inactivity caused by the pandemic, though not directly measured may have influenced performance trajectories, as observed in other Paralympic athletics events. According to Marmeleira et al. (28), performance trends in athletes with visual impairment have shown consistent improvements across eight Paralympic cycles, indicating that with structured development, athletes can recover from disruptions and continue to advance.

From 2019 to 2021, an improvement in marks for all classes was seen in the international ranking, which may hypothetically reflect the implementation of individualized training strategies, periodization adjustments, and a focused peaking strategy directed towards the Tokyo 2020 Paralympic Games. This assumption aligns with findings in masters’ athletics, where female athletes have displayed steady performance improvements across calendar years, particularly in throwing events. Kundert, Di Gangi, et al. (9) found that, among older female athletes in power-based disciplines, accumulated training and technical refinement contributed more to performance maintenance than age itself.

Interestingly, such trends may also indicate a narrowing of the gender performance gap in Paralympic contexts. Long-term studies of jumping and throwing disciplines (27) have demonstrated a modest reduction in gender disparities in sprint events between 1992 and 2012, especially in classes with higher impairment levels. Likewise, Knechtle et al. (34) found evidence of convergence in male and female performance in endurance and strength events, potentially due to improved access to coaching and training conditions.

To identify a strategic class for winning Paralympic medals, NPCs should consider class F11, which exhibited a positive accumulated performance evolution (%Ac = 2.07%) for the analyzed period, indicating sustained growth despite external constraints. The class exhibited the lowest improvement in technical performance among the Top 3 groups. This class represents a potentially favourable target for national investment, particularly given that performance progression has been shown to vary according to sports class and severity of impairment. Marmeleira et al. (28) highlighted that athletes with greater impairment severity (e.g., T/F11) may experience slower initial development but demonstrate significant relative gains over time, underscoring the importance of targeted long-term planning.

The process of attracting new athletes within Paralympic sport is influenced by the origin of the athlete’s impairment, whether congenital or acquired, and factors that may be associated with athletic success in specific para sports. The developmental journey toward high performance is inherently tied to the classification process, which determines the sport class in which the athlete will compete, in accordance with the minimum eligibility criteria. From that point forward, the athlete and coach work collaboratively to achieve results that enable participation across various competitive levels.

However, it is essential to emphasize that studies such as Piacentini et al. (30), analyzing Olympic throwing events, revealed high dropout rates even among finalists at World Junior Championships, ranging from 28% to 58%, depending on the event and year, highlighting the critical need for long-term planning and appropriate management of early specialization (30). This is particularly relevant to throwing events in women’s Para athletics, which require sustained technical development and often receive less structural support in the early stages.

Given the specific dynamics of Paralympic sport, the proactive identification of sport classes with investment potential has become increasingly emphasized in literature. Although coaches can effectively lead athlete development, relying solely on coaching intuition may limit strategic planning. Instead, performance monitoring across key competitive cycles such as the Paralympic, World, and continental events should be adopted as evidence-based practice. Grobler et al. (27) showed that long-term performance improvements are not linear but often follow class-specific trajectories that reflect the structure of competition, training access, and technical demands.

It is essential to highlight that athletic progression should not be interpreted as a continuous linear process. Often, annual averages did not differ significantly from the previous year, indicating that the evolution of results is modulated by the functional classification system adopted for international competitions. This observation aligns with the findings of, who demonstrated that fluctuations in performance are influenced by the competitive structure and classification criteria, rather than only by athlete development.

To identify optimal sport classes and align athlete profiles with eligibility criteria, classifiers must play an active role in the recruitment and talent development process. According to Marmeleira et al. (28), athletes with severe impairments, such as those in F11 classes, often show slower initial performance gains but may experience substantial long-term progress when appropriately supported from an early stage.

Integrating classifiers and data analysts into the athlete identification system enhances the ability to detect functional profiles aligned with international medal potential, especially when combined with tools that assess intra- and inter-class performance dispersion. As Frossard (8) argued, understanding the variability within and between sport classes is fundamental to ensuring fair classification outcomes and optimizing talent identification strategies.

Therefore, understanding the athlete’s performance pathway and the key factors that influence retention is well established in literature as an essential strategy to support the transition to elite competition. To maximize this process, it is necessary to combine longitudinal analysis of performance trends by sport class with an understanding of athlete profiles and eligibility rules, and to disseminate this knowledge to coaches, clubs, and rehabilitation centers.

Given the specific nature of the Paralympic classification system, tracking the progress of results across various time frames (such as Paralympic cycles, continental competitions, and World Championships) has become a well-established approach in scientific literature. While coaches can steer athlete development through training periodization, incorporating technical analyses of performance trends improves talent identification processes. Therefore, functional classifiers should actively participate in identifying classes with medal potential, ensuring that this knowledge is shared with clubs and institutions involved in Paralympic sport development.

## 5 Conclusion

This study identified key performance trends in men’s and women’s discus throw events in Paralympic athletics over the 2012–2022 period. Notable fluctuations were observed, particularly around the 2020 season, due to the COVID-19 pandemic and a disrupted qualification cycle. The 2017–2020 cycle did not permit the construction of a consistent four-year performance curve, thereby limiting longitudinal analysis. Nevertheless, some classes demonstrated performance recovery by 2021, highlighting the resilience of elite para-athletes despite the effects of detraining and reduced competition exposure.

The findings underscore the importance of long-term, class-specific monitoring to guide talent development and strategic investment. Variations in accumulated performance across classes, such as the decline in F37 (men) and growth in F11 (women), reveal the need for integrated planning among coaches, classifiers, and analysts. Such collaboration can enhance athlete retention and improve medal prospects, particularly when external factors disrupt performance trends.

## Data Availability

Publicly available datasets were analyzed in this study. This data can be found here: https://www.paralympic.org/athletics.

## Footnotes

^1^Sport classes follow the *World Para Athletics* classification system. Class F11 includes athletes with severe visual impairment, characterized by total or near-total absence of light perception, who compete with mandatory eye shades and the assistance of a sport assistant. Classes F37 and F38 comprise athletes with coordination impairments of neurological origin, such as cerebral palsy, with F37 characterized by unilateral impairment (hemiplegia) and F38 by mild to moderate impairment with better functional control. Class F41 is designated for athletes with short stature resulting from skeletal dysplasias. Classes F52, F53, F55, and F56 include athletes who compete seated in a throwing chair, with varying levels of trunk and lower-limb impairment, ranging from minimal trunk control (F52) to moderate trunk function (F56). Finally, class F64 comprises athletes with lower-limb impairment who compete standing using a prosthesis.
